# Crystal shape controlled H_2_ storage rate in nanoporous carbon composite with ultra-fine Pt nanoparticle

**DOI:** 10.1038/srep42438

**Published:** 2017-02-14

**Authors:** Tsan-Yao Chen, Yanhui Zhang, Liang-Ching Hsu, Alice Hu, Yu Zhuang, Chia-Ming Fan, Cheng-Yu Wang, Tsui-Yun Chung, Cheng-Si Tsao, Haw-Yeu Chuang

**Affiliations:** 1 Department of Engineering and System Science, National Tsing Hua University, Hsinchu 30013, Taiwan; 2 Institute of Nuclear Engineering and Science, National Tsing Hua University, Hsinchu 30013, Taiwan; 3 Department of Mechanical and Biomedical engineering, City University of Hong Kong, 88 Tat Chee Ave, Kowloon, Hong Kong, SAR; 4 Scientific Research Division, National Synchrotron Radiation Research Center, Hsinchu 300, Taiwan (R.O.C.); 5 Industrial Technology Research Institute, Green Energy and Environment Research Laboratories, Hsinchu 310, Taiwan; 6 Department of Materials Science and Engineering, Feng Chia University, Taichung 40724, Taiwan; 7 Institute of Nuclear Energy Research, Atomic Energy Council, Executive Yuan, Taoyuan City 32546, Taiwan; 8 Green Technology Research Institute, CPC Corporation, Taiwan, Kaohsiung 81126, Taiwan, R.O.C

## Abstract

This study demonstrates that the hydrogen storage rate (HSR) of nanoporous carbon supported platinum nanocatalysts (NC) is determined by their heterojunction and geometric configurations. The present NC is synthesized in an average particle size of ~1.5 nm by incipient wetness impregnation of Pt^4+^ at carbon support followed by annealing in H^2^ ambient at 102–105 °C. Among the steps in hydrogen storage, decomposition of H_2_ molecule into 2 H atoms on Pt NC surface is the deciding factor in HSR that is controlled by the thickness of Pt NC. For the best condition, HSR of Pt NC in 1~2 atomic layers thick (4.7 μg/g min) is 2.6 times faster than that (1.3 μg/g min) of Pt NC with higher than 3 atomic layers thick.

Hydrogen is one of the most effective energy forms in terms of power density and conversion efficiency for keeping human civilization sustainable. However, in current development of technologies, the capacity of hydrogen storage is often too low to be used as a daily energy supply, except for carbon supported Pt nanocatalyst (NC). This material has been classified as a potential means to the on-board hydrogen storage material according to the Department of Energy (DOE), U.S.A. The hydrogen storage capacity of this material is influenced by chemical and physical properties in both carbon support (defect density, ligand identity, porosity) and the supported NC (composition, shape, and configuration). Among existing pathways, the spillover is considered to be a predominate mechanism in decomposition and subsequent storage of hydrogen in the interfaces of Pt NC^1^,^2^. Although many studies and applications have been devoted to spillover mechanism, the difficulty to reproduce the earlier results indicates that gaps between experiment and fundamental theory in heterogeneous catalysis still exist^3^,^4^,^5^.

Our previous work adopted inelastic neutron scattering analysis to reveal the fundamental pathways for H_2_ storage on Pt NC^6^,^7^,^8^. On Pt NC surface, hydrogen molecules are dissociated into hydrogen atoms that are either bound to neighboring defect sites or trapped by pi-electron resonance on surface of the nanoporous carbon. A complementary XPS analysis shows that formation of C-H bonds results in 1.2–1.5% mass fraction of hydrogen storage on Pt decorated single walled carbon nanotube^9^.

Although the spillover pathways are getting clear, little is known about the impacts of geometric configuration of nanoporous carbon supported Pt clusters on H_2_ storage rate (HSR)/capacity. To elucidate the effects of crystal shape on HSR of Pt/AC, this study employs several methods, including small angle X-ray scattering (SAXS), X-ray absorption spectroscopy (XAS), X-ray diffraction (XRD), and density functional theory calculation (DFT). Our finding shows that the rate of H_2_ decomposition is controlled by the crystal thickness of carbon supported Pt NC. For the best condition, HSR of Pt NC in 1~2 atomic layers thick (4.7 μg/g min) is 2.6 times faster than that (1.3 μg/g min) of Pt NC with higher than 3 atomic layers thick. Details of configuration and surface energy determination in relation to the HSR of Pt/AC are given in the following sections.

## Results

From theoretical geometrical calculation, surface-to-bulk ratio of NC with a known size is proportional to the crystal shape. The step-by-step strategy for determining the impacts of crystal shape on HSR of Pt NC is (1) XRD and SAXS analyses on the coherent length and geometrical asymmetry of NC in carbon support, (2) surface-to-bulk ratio determination by cross-referencing results of XAS and geometrical calculation, and finally (3) adsorption energy calculation on Pt-H bondings at Pt NC with thickness determined by structure characterizations.

### Crystal structure and nanostructure of active carbon supported Pt nanocatalyst (Pt NC)

**Figure 1:**
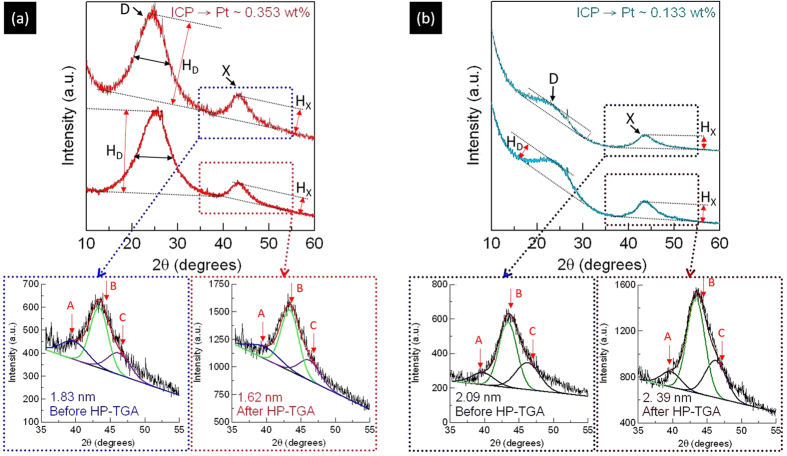
XRD patterns of Pt doped nanoporous active carbon annealed at (**a**) 105 °C and (**b**) 130 °C. The spectra were collected before and after HP-TGA.

Crystal structure of Pt/AC and carbon support are revealed by using XRD analysis. Figure 1 demonstrates the XRD pattern of INER_A (1a) and INER_B (1b) before and after conducting the H_2_ purged High-pressure Thermogravimetric analyzer (HP-TGA). As indicated in Fig. 1, peak D is the characteristic line of (002) facet for active carbon (AC) while peak X represents the lines of (111)/(220) facets for metallic Pt and (110) facet for PtO (or (002) facet for PtO_2_). For freshly prepared NC, the average coherent length (h_avg_) of metallic phase at Pt (111)/(200) facets is 9.2/10.2 Å for INER_A and 11.1/11.2 Å for INER_B. After HP_TGA treatment, h_avg_ of Pt(111)/Pt(200) in INER_A is decreased/increased by 17.4%/11.8%. In INER_B, h_avg_ of Pt(111)/Pt(200) is increased by 8.1/9.8% after exposed to the same treatment. In this condition, h_avg_ of Pt metal oxide phase is decreased by 9.0% in INER_A and is increased by 10.7% in INER_B.

**Figure 2:**
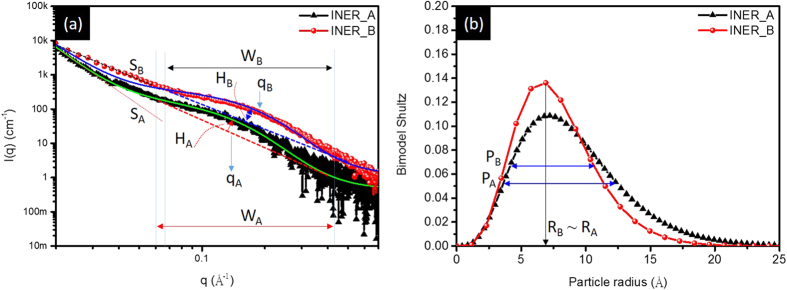
(**a**) Small angle X-ray scattering spectra of Pt cluster doped nanoporous carbon materials and (**b**) corresponding particle Bi-model Shultz size distribution of supported Pt clusters.

Average particle size (D_avg_), particle size distribution (P_R_), and particle shape of Pt NC are characterized by SAXS analysis. In general, features of SAXS spectra result from the interference between incident X-ray and both inter-particle (Q < 0.07 Å^−1^) and intra-particle (Q > 0.07 Å^−1^) structures. Inter-particle structure that is also called structure factor (S(Q)) include inter-particle distance as well as the size and packing density of inter-particle agglomerates. Known as form factor (P(Q)), intra-particle structure consists of the shape, dimension, and configuration of NC. This study only focuses on the discussions related to P(Q) of SAXS spectra because of the dominant role of intra-particle structures in H_2_ decomposition activity of NC. Figure 2a compares the SAXS spectra of the experimental Pt/AC with least-square fitting curves calculated by the model of polydispersed spheres with bimodal Schulz distribution (ESI); where background scattering of pure AC powder was subtracted for extracting the contribution of metallic NCs. In the low q range, in comparison with INER_B, the SAXS spectrum of INER_A shows a higher scattering intensity gradient (denoted by slope of S_A_), which indicates a larger surface roughness (or asymmetry) of NC in INER_A. In the high q region, the scattering hump at q_i_ with a width (W_i_) and a height (H_i_) results from scattering interferences of among X-ray and intra-particle structures of NC. The ratio of W_i_/H_i_ is proportional to surface roughness and polydispersity (P_i_) of NC; subscripts A and B denote respectively parameters of INER_A and INER_B.

In INER_A spectrum, as compared to that of INER_B, shift of oscillation hump (from q_B_ to q_A_) to low q and reduction of W_i_/H_i_ ratio indicate a larger geometrical asymmetry of NC. Figure 2b shows particle size distribution (Schulz distribution) of experimental Pt NCs. Accordingly, the average radius of both INER_A and INER_B is ~6.9 Å (i.e., D_avg_ ~ 14 Å). Compared to P_B_, a broadened P_A_ (~35.2%) with an asymmetrical profile (tailing to high R region) implies a high aspect ratio of NC in INER_A.

### Chemical composition and surface oxidation ratio of Pt NC affected by pre-metal doping treatment on active carbon support

**Figure 3:**
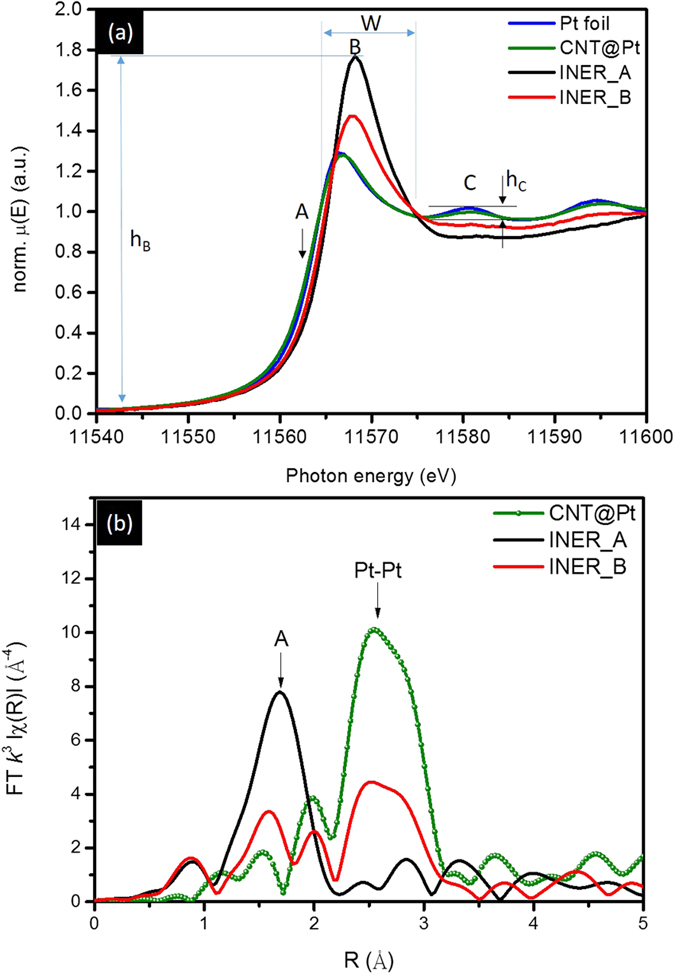
Pt L_3_-edge (**a**) X-ray absorption near-edge spectra and (**b**) extended X-ray absorption fine structure spectra of Pt cluster doped nanoporous carbon materials and CNT supported Pt NP.

Figure 3a compares Pt L_3_-edge X-ray absorption near-edge spectra (XANES) of experimental Pt NCs and standard samples (CNT supported Pt NC and Pt foil). In a L_3_-edge spectrum, the inflection point position (i.e., 1^st^ deviation maximum (E_0_) at arrow X of XANES spectra in Fig. S1) refers to the minimum energy of photoelectron for initiating electron transitions from 2p_3/2_ to 5d_5/2_ orbitals. Intensity (h_B_) and width (W) of near-edge absorption peak (arrow B) denote respectively the 2p_3/2_ to 5d_5/2_ transition probability and band width of 5d orbital of Pt atoms. As depicted in Fig. 3a, the strongest h_B_ shows the highest p-d transition of Pt atoms in INER_A among all the samples. In the meantime, flattened post-edge hump (i.e. h_C_/W_C_ ratio) refers to local disordering of severe Pt oxidation in INER_A. On the other hand, in INER_B, a significantly decreased h_B_ accounts for the presence of metallic Pt clusters. Chemical composition of experimental Pt NCs is further illustrated by linear combination fitting (LCF) method. As shown in Table 2, Pt oxide (PtO and PtO_2_) ratio is 79.7% in INER_A and 42.9% in INER_B.

Figure 3b compares Fourier transformed EXAFS spectra (i.e., radial functions) of experimental Pt NCs and CNT supported Pt. Accordingly, Pt oxide and metal characters are evident in INER_B as revealed respectively by the radial peak ranging from 1.0 to 1.75 Å (Pt-O bond pair) and that from 2.2 to 3.15 Å (Pt–Pt^M^ bond pair). The results of model analysis (Table 3) show that the coordination number (CN) of Pt oxide (Pt-O, CN_Pt-O_) is 1.31 (R_Pt-O_ = 1.922 Å) and that of metallic Pt-Pt bond (CN_Pt–Pt_) is 2.01 (R_Pt-Pt_ = 2.754 Å). In INER_A, peak A results from the X-ray interference with Pt-O1 (1.922 Å) and Pt-O2 (2.079 Å) bonds. In the event, CN of Pt-O1 is 1.86 and that of Pt-O2 is 2.87 for PtO_2_ phase in experiment NC. Compared to CN of ideal PtO_2_ crystal, CNs of Pt-O1 and Pt-O2 are both reduced but to different extent. CN_Pt-O1_ decreases by ~7% while CN_Pt-O2_ falls by ~28.2%. The uneven reduction explains the growth of asymmetrical 2D Pt crystal in INER_A.

### Surface-to-bulk ratio determination by chemical composition of Pt NC

**Figure 4:**
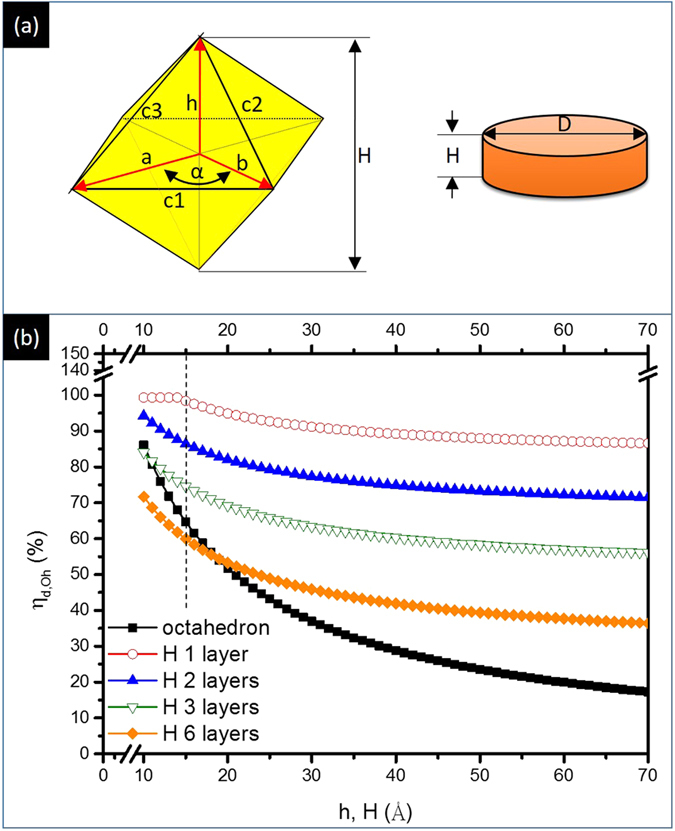
(**a**) Schemes for octahedron (h, a, b, c1, c2, and c3 denote the height, two axis in horizontal plan, and side lengths of triangle planes in octahedron) and disk-like (H and D denote the thickness and diameter of disk crystal) and (**b**) surface to bulk ratio (η) of octahedron and disk like crystals as a function of h or H, where H 1, 2, 3, 6 layers denote η of disk crystal with corresponding atomic layer in thickness.

To confirm crystal shape, the surface-to-bulk ratio (η) of experimental NCs is compared with that of ideal NCs in different geometries (scheme shown in Fig. 4a). Eqn 1 and eqn 2 represent the estimation for η of ideal NCs that changes by varying thickness (disk-like crystal, η_d_) and height (octahedron crystal, η_Oh_)^10^.


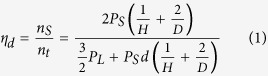



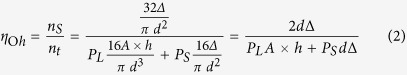


In eqn 1, factors of d, P_S_, P_L_, H, and D respectively stand for the diameter of atom, surface atomic packing factor, bulk atomic packing factor, thickness of NC, and diameter of NC. In eqn 2, factors of Δ, A, and h represent respectively area of triangle in outmost surface, area of triangle in horizon plane, and height of octahedron. In Fig. 4b, η_d_ for H = 1 NC is exponentially decreased by 13% (100 to 87%) with D from 10 to 70 Å. In the cases of H = 2 and 3 NCs, η_d_ is decreased by 18% (94 to 72%) and 31% (88 to 57%), respectively. These values are higher than 50%, indicating the lack of bulk atom in disk NC when H ≤ 3^11^,^12^. For octahedron NC, bulk atom ratio goes up significantly by 68% (i.e., η_Oh_ is decreased from 87 to 19%) with an increases of h from 10 to 70 Å. Taking physiochemical natures into consideration, oxidation occurs only in the first two atomic layers from the outmost surface of Pt NC in an ambient condition below 150 °C. This hypothesis suggests the extent of oxidation for Pt NC is a function of its η. In this study, one can notice that D_avg_ of nanocrystallite is ~14 Å for both INER_A and INER_B, where Pt NC is preferentially grown in 2D clusters and 3D ones, respectively. Consequently, taking results of Table 1 and Fig. 4b into account, the substantial high η (79.8%) of INER_A implies the growth of 2D Pt NC in a H ranging from 1 to 2 atomic layers. In INER_B, η_B_ is 42.9%, indicating the growth of 3D Pt clusters. The value of η_B_ is slightly decreased from that of octahedron crystal with a h = 14 Å. This result could be attributed to the growth of Pt clusters in polymorph whose surface facets are more than those of octahedron crystal.

### Density functional theory calculation on impacts of crystal shape to H_2_ storage rate on Pt NC

**Figure 5:**
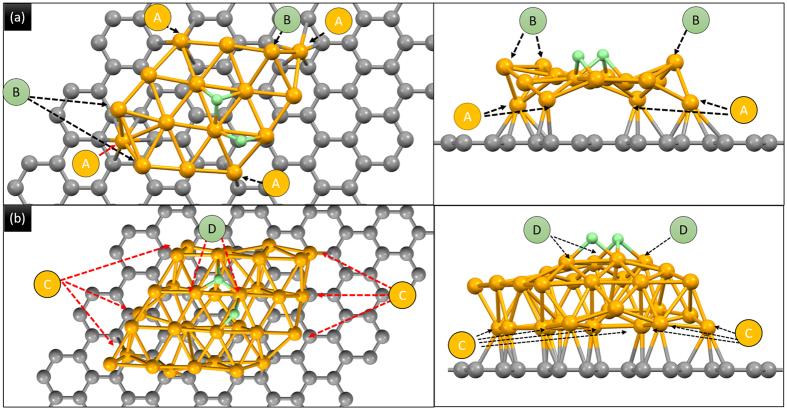
DFT simulated atomic structures of two H atoms adsorbed on sorption sites of (**a**) one atomic layer Pt on carbon (1PtAC) and (2) 2 atomic layers of Pt on carbon (2PtAC).

In order to determine the optimal structure, different configurations of monolayer Pt atoms placed at different stacking sites on graphite surface are built. Configuration with Pt atom on atop position is named as 1PtAC, on bridge site as 1PtB, on hollow sites 1PtC, and 1PtAC for pairs at both atop and hollow sites. Average distance between Pt atom and graphene layer is 2.32 Å. The packing structure and energy relaxation profile of two of those models are shown in Fig. S2a and S2b. Among all configurations, 1PtAC model possesses the lowest binding energy (E_dep_) of ~0.33 (eV/Pt atom). Therefore, effects of Pt thickness on the H_2_ binding energy in Pt NC surface are further investigated by DFT calculation with monolayer (1PtAC in Fig. 5a) and two atomic layers (2PtAC in Fig. 5b) of Pt on graphene. In simulation, position of carbon positions is fixed to reproduce the bonding characteristics between bulk graphite and Pt atoms. As depicted in Fig. 5, Pt atoms at corner (arrows A) tend to seat in hollow sites with 2–4 bondings with carbon layer. The neighboring Pt atoms which are at high z position (arrows B) indicate the Pt-Pt homoatomic bonding distinguished from Pt-C. Rest of the Pt atoms kept at the original position with a displacement of +/− 0.05 Å in height. H atoms are located at hollow and bridge sites on Pt layer with a bond length of 1.540 and 1.750 Å, respectively. The rough Pt surface of 1PtAC implies a high density of dangling bond which is in consistent to the result from spectrum analysis that carbon oxidation supports 1 to 2 atomic layers Pt cluster. For 2PtAC, all Pt atoms at interface form multiple bondings with carbon atoms (arrows C) and atoms tend to agglomerate in 3D cluster as a result of the strong Pt-Pt bond. In this structure, the center Pt atoms are lifted up by 0.1 Å or more in z direction (arrows D) and bond length of Pt-H is 1.804/1.980 Å in hollow sites and 1.742/1.836 Å in bridge sites. H atoms locate at bridge and hollow sites of Pt layer where corresponding atoms are at hollow and atop sites of graphene underneath.

Calculation results are given in Table 4. Accordingly, cohesive energy (E_c_) of 1PtAC is 0.582 eV stronger than that of 2PtAC with a H_2_ molecule in vacuum. This result accounts for the stronger attraction force to molecular H_2_ in 2D Pt cluster as compared to that of 3D Pt ones. After relaxation, H atoms are preferentially located at hollow and bridge sites of 1PtAC and at hollow sites of 2PtAC. The coherence energy difference (ΔE_c_ = E_c_^f^ − E_c_) between initial and final state is resulted from formation of surface Pt-H bond and is −0.069 (eV Pt atom^−1^) for 2PtAC and −0.255 for 1PtAC (eV Pt atom^−1^). There are two factors for estimation of E_c_ including (i) number of Pt atom and (ii) distance/bond strength between H and Pt atoms. In the first factor, ΔE_c_ should be doubled by reducing 50% of Pt atoms in DFT model. However, ΔE_c_ of 1PtAC is 2.7 times higher than that of 2PtAC. Such an abnormal ΔE_c_ could be accounted for the formation of strong Pt-H bond due to the coupling effects between adsorbed H on Pt atom and pi-electron in hollow sites of carbon substrate underneath. Adsorption energy (E_a_) for H atom on sorption site of 1PtAC (1PtAC-2H model) is -2.043 (eV atom^−1^) and 2PtAC-2H is −1.117 (eV atom^−1^).

## Discussion

Crystal shape of NC is one of the most influential factors in its HSR because different atomic packing densities of crystal facets will change the activation energy for redox reactions. For nanocrytallite with a known size, surface-to-bulk ratio (η) is proportional to its crystal shape. The ratio can be estimated by oxidation ratio considering that surface chemisorption of oxygen is a general form of oxide in the outmost layer of Pt NC. Therefore, in determining the correlation between crystal shape to HSR of Pt NC, one should confirm their particle size, oxidation state, and the structure asymmetry by structure characterizations. After that, results of theoretical model calculations on η to crystal shape is needed. In this study, X-ray diffraction (XRD), small angle X-ray scattering (SAXS), X-ray absorption spectroscopy (XAS) analyses, and theoretical geometry calculation are employed to reveal the bulk structure parameters, oxidation ratios, as well as η in relation to the shape of Pt NC. To investigate the impacts of crystal shape on HSR of Pt NC, density functional theory (DFT) calculation on proper atomic models is performed.

**Figure 6:**
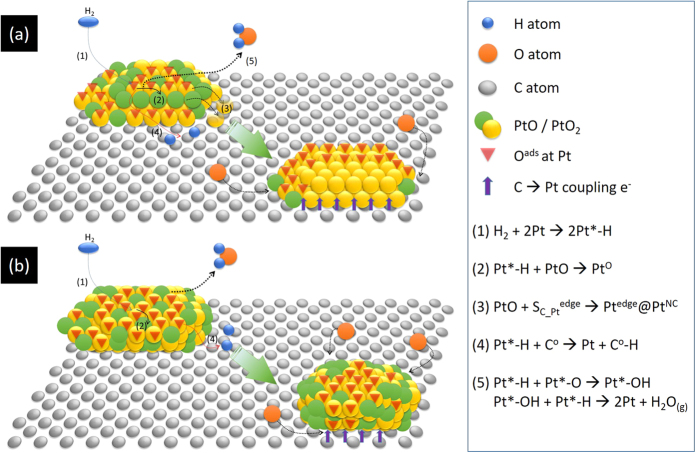
Schematic representations to Pt atom restructure in HP-TGA analysis for (**a**) INER_A and (**b**) INER_B.

In XRD analysis, ratios of peak X and peak D intensity (H_X_/H_D_) are substantially increased from 0.28 (INER_A) to 1.67 (INER_B), which indicates the increasing of long-term ordering structure in carbon support by decreasing Pt content from 0.35 to 0.13 wt%. After HP-TGA test, the further decreased H_X_/H_D_ implies a disruption of long-term ordering in carbon support. This phenomenon results from intercalation of Pt clusters into carbon support by different extents between INER_A (22.2%) and INER_B (35.4%). In this event, decreasing Δ(H_X_/H_D_) with increasing full width at half maximum (FWHM) of peak D depicts a restructure of Pt atoms into low-dimension Pt clusters on the high dangling bond density surface of INER_A. This hypothesis is further confirmed by uneven changes between average coherent length (h_avg_) of Pt facet (111) (decreased by 17.8%, from 9.2 to 7.6 Å) and (200) facet (increased by 11.8%, from 10.2 to 11.4 Å). Such a phenomenon could be attributed to relocation of atop Pt atoms to carbon sites around metal clusters (Fig. 6).

In the meantime, h_avg_ of Pt oxide phase is decreased by ~9.0% revealing weak Pt-O bonding on Pt atoms directly adjacent to electron rich support (i.e., shielding effects of graphite or graphene). The growth of 2D Pt cluster is further confirmed by the asymmetry SAXS scattering profile of INER_A. On the other hand, h_avg_ of Pt metal and Pt oxide phases in INER_B is increased by 8.1~10.7% after HP-TGA. The growth of both metal and oxide phases is attributed to Pt restructure into 3D cluster by inter-particle coalesce as a result of weak attachment of NC at carbon support. In this event, Pt-O phase is formed in 3D Pt NC surface.

From SAXS analysis, D_avg_ (~1.4 nm) of Pt NCs in INER_A is similar to that of INER_B. Based on XRD analysis, h_avg_ of Pt NCs in the two samples is highly dependent on surface-to-bulk ratio. These observations validate the correlation between η and shape of NC with a known size. To identify the crystal shape of experimental NCs, correlations between their oxidation state and η are investigated by cross-referencing the results of atomic arrangement, oxidation states, and geometry configurations (VEDO method)^10^. In INER_A, the content of Pt metal is 20.3% and that of Pt oxide (PtO and PtO_2_) is 79.7%. Given that surface oxygen chemisorption is a general form in Pt NCs, their oxidation ratio also refers to surface-to-bulk ratio. Accordingly, η of INER_A (η_A_) is 79.7%. This value is higher than η_Oh_ of a 1.4 nm octahedron (62%), suggesting the growth of disk-like Pt NCs in a thickness between 1–2 atomic layers. As for INER_B, η_B_ is 42.9% indicating the formation of 3D Pt NCs.

Based on the results of structural analyses and H_2_ sorption test, our study shows that Pt atoms tend to stack into 2D NC on active carbon by annealing at 102 °C under H_2_ for 2 h (hydrogen reduction) after a metal ion impregnation process. In this condition HSR of carbon supported Pt NC is 4.7 μg/g min. By increasing annealing temperature to 130 °C under H_2_, Pt atoms are shaped into 3D polymorph NC on active carbon; the NC HSR becomes 1.3 μg/g min. To clarify the impacts of crystal shape on HSR of Pt NC, H_2_ sorption energy of proper atomic stacking scheme is simulated by *ab initio* DFT calculation. Our hypothesis is further confirmed by the results of binding energy of H atom (E_a_) in current models. As indicated in Table 4, the endothermic E_a_ indicates that the spillover decomposition of H_2_ molecule into 2 H atoms on Pt surface and positioning of the H atoms to neighboring sites are thermodynamically spontaneous reactions in 1PtAC-2H (E_a_ = −2.043 eV atom^−1^). In 2PtAC-2H, E_a_ is −1.117 (eV atom^−1^). The smaller E_a_ suggests a weaker Pt-H bonding in 2PtAC as compared to that of 1PtAC. It also means a higher diffusion coefficient of H atoms, which indicates a strong recombination tendency of adsorbing H atoms into H_2_ molecule and thus the suppressed HSR in 2PtAC. Taking together, our results of DFT calculation and structure characterizations explain that geometrical configurations of Pt NC is a determining factor in both HSR and H_2_ storage capacity of metal doped nanoporous carbon materials^6^,^7^,^8^.

In short, our findings illustrate that Pt atoms tend to aggregate into disk-like NCs with a thickness of 1~2 atomic layers in properly selected carbon support (SSA = 1886 m^2^ g^−1^). By increasing the hydrogen-reduced temperature to 130 °C and the use of carbon support without acid (function group) treatment, nanoscaled polymorph Pt clusters in 3D are formed on carbon support. We adopt structural characterizations (XRD, SAXS, XAS) and theoretical model calculations to illuminate that HSR for Pt doped nanoporous carbon materials is dominated by the crystal shape of supported Pt NC. In a 2D cluster with a thin atomic layer, H_2_ molecule will decompose into 2 H atoms. After that, H atoms will be adsorbed in atop sites in the rough surface. In contrast, in a 3D cluster, H atoms tend to locate in hollow sites of Pt surface with a substantially reduced E_a_ as compared to that of a 2D cluster. Therefore, the HSR of a 3D Pt cluster is drastically lower than that of a 2D cluster.

## Methods

### Synthesis and the gravimetric hydrogen uptake measurement of Pt doped nanoporous activated carbon composite (Pt NC)

The Supporting Material for growing Pt clusters is a nanoporous active carbon (ACB16) with a BET specific surface area (SSA) of 1886 m^2^g^−1^. The Pt clusters were synthesized by incipient wetness impregnation of Pt^4+^ ions in ACB16 with a vacuum-attraction system^13^,^14^. The subsequent hydrogen reduction was performed at 102 °C (rising rate = 5 °C min^−1^) for 2 hours. To reinforce NC attachment, the carbon support was washed in an acid base before Pt impregnation. In this treatment, 10 g nanoporous carbon powder was dispersed in the 500 mL acid solution of H_2_SO_4_ (95%), HNO_3_(65%) and distilled water The mixed solution was heated from room temperature to 105 °C (rising rate = 0.2 °C min^−1^) and then cooled to room temperature. Details for the experiment procedures are given in our previous works^14^. The samples were named as INER_A (Pt cluster on freshly prepared ACB16) and INER_B (Pt clusters grown on the un-acid-treated carbon ACB17 with SSA = 1857 m^2^g^−1^ was hydrogen-reduced 130 °C for 2 hours) which respectively contained 0.35 and 0.11 wt% of Pt metal (Table 5). The gravimetric HSRs for the experimental Pt/AC at room temperature were measured by using HP-TGA (Cahn-Thermax-500) of Institute of Nuclear Energy Research, Taiwan). The parameters for preparing the two samples were decided in resulting their optimum HSR. Details of measurement receipt are similar to those given elsewhere^14^. The small angle X-ray scattering (SAXS) spectra were measured using the Bruker Nanostar instrument^6^. The shape of NC was determined by combing the results of Pt oxidation state and the surface ratios of nanocrystallites that vary according to the changes of their sizes in a 3D atomic packing model (VEDO)^10^.

### Density Functional Theory Calculation

To investigate the H-adsorption on Pt cluster decorated graphite structure, we have carried out first-principles calculations using Vienna Ab initio simulation package (VASP)^15^,^16^. The calculations were conducted at formosa5 cluster of National Center for High Performance Computing (NCHC), Taiwan. We adopted the projected augmented wave (PAW)^17^,^18^ pseudopotential method with local density approximation, in which plane-wave energy cutoff of 600 eV and Monkhorst–Pack *k*-point mesh^19^ density of 0.03 Å^−1^ are used for all calculations. To explore the optimized decoration sites of nano-Pt on graphene, the atop (A), bridge (B), hallow (C) and A&C mixing sites, and decoration distance from 2.1 Å to 3.7 Å were tested. For Pt-decoration sites at atop (A), bridge (B), hallow (C), the supercell’s lattice size was fixed at a = b = 19.68 Å with the optimized C-C bond length of 1.42 Å (128 C + 16Pt atoms). For the mixing decoration sites of A&C, another supercell was built up with the lattice size fixed at a = 17.0434, and b = 25.5651 Å, which ensures the C-C bond length kept at 1.42 Å (144 C + 16 Pt atoms). All the supercells were built up with at least 24 Å vacuum regions in the z-direction to avoid the interaction between adjacent periodic images. To find the most energetically favorable structures, all the Pt atoms were fully relaxed with the supercell size and graphene atoms fixed (stiff graphene without topological change). The ionic relaxation was stopped when the total energy change is below 1 meV.

## Additional Information

**How to cite this article**: Chen, T.-Y. *et al*. Crystal shape controlled H_2_ storage rate in nanoporous carbon composite with ultra-fine Pt nanoparticle. *Sci. Rep.* **7**, 42438; doi: 10.1038/srep42438 (2017).

**Publisher's note:** Springer Nature remains neutral with regard to jurisdictional claims in published maps and institutional affiliations.

## Supplementary Material

Supplementary Information

## Figures and Tables

**Table 1:** XRD determined crystal structure parameters of Pt cluster doped nanoporous carbon materials.

Sample	Peak	HP-TGA*	2θ	Index	Δ2θ red	h_avg_ (Å)	Δh_avg_ (%)	d (Å)	H_X_/H_D_ and Δ (H_X_/H_D_)	
INER_A	A	X	39.7	Pt (111)	4.779	9.2	−17.4	2.28	H_X_/H_D_-X	0.28
		O	39.3	Pt (111)	5.808	7.6		2.3	H_X_/H_D_-O	0.22
	B	X	43.2	PtO (110)/PtO_2_(002)	3.345	13.4	−9.0	2.1	Δ (H_X_/H_D_)	22.2%
		O	43.2	PtO (110)/PtO_2_(002)	3.676	12.2		2.1		
	C	X	46	Pt (200)	4.522	10.2	11.8	1.98		
		O	46.3	Pt (200)	3.97	11.4		1.97		
INER_B	A	X	39.4	Pt (111)	3.97	11.1	8.1	2.3	H_X_/H_D_-X	1.67
		O	39.5	Pt (111)	3.676	12		2.29	H_X_/H_D_-O	1.08
	B	X	43.4	PtO (110)/PtO_2_(002)	3.67	12.2	10.7	2.1	Δ (H_X_/H_D_)	35.4%
		O	43.2	PtO (110)/PtO_2_(002)	3.309	13.5		2.11		
	C	X	46.3	Pt (200)	4.04	11.2	9.8	1.97		
		O	46.1	Pt (200)	3.676	12.3		1.98		

^*^X: before HP-TGA test, O: after HP-TGA test. H_X_/H_D_-X and H_X_/H_D_-O denote the H_X_/H_D_ value before and after HP_TGA test.

**Table 2:** Linear combination fitting method determined chemical composition of Pt cluster doped nanoporous carbon samples Pt/ACs.

Sample	ΔE (eV)*	Pt (%)	Pt^II^O (%)	Pt^IV^O_2_ (%)
INER_A	1.75	20.3 (4.1)	37.5 (4.6)	42.1 (6.1)
INER_B	0.70	57.1 (1.7)	17.1 (1.7)	25.8 (2.4)

^*^ΔE: the energy difference of first deviation maximum between samples and standard Pt foil.

**Table 3:** EXAFS model simulated atomic structure parameters of Pt cluster doped nanoporous carbon samples (Pt/ACs) and CNT supported Pt NP (CNT@Pt).

Sample	Path	CN	CN ideal	R (Å)	ΔR (Å)
CNT@Pt	Pt-O	0.60	2	1.969	0.054
	Pt-Pt	6.14	12	2.726	−0.046
INER_A	Pt-O1	1.86	2	1.972	0.056
	Pt-O2	2.87	4	2.079	0.056
INER_B	Pt-O1	1.31	2	1.922	0.006
	Pt-Pt*	2.01	12	2.754	−0.018

^*^Pt-Pt bond pair of metallic Pt fcc phase.

**Table 4:** DFT calculation determined energies of H_2_ mole and H atoms in NP surface containing different atomic layers of Pt atom in top of graphite structure.

Model	E_c_^**^ (eV)	E_c_^f ***^ (eV)	E_a_ (eV)	RPt-Hh (Å)	RPt-Hb (Å)
	H_2_ in vacuum	H_2_ in sorption site	Pt-H at sorption site		
1PtAC	−1.767	−2.023	−2.043	1.540	1.740
2PtAC	−1.185	−1.255	−1.117	1.804/1.980	1.742/1.836

*For mode with lowest energy, H adsorbed in atop sites of 1PtAC and hollow sites in 2PtAC; E_c_: cohesive energy of model; E_c_^f^: cohesive energy of model for H adsorbed PtAC slab after relaxation; E_a_: sorption energy of Pt-H in sorption sites; RPt-Hh: Pt-H bond length (H at hollow site); RPt-Hb: Pt-H bond length (H at bridge site).

**Table 5:** Physical Characteristics and hydrogen storage rate (HSR) of experimental Pt doped nanoporous carbon materials.

Sample	Pt treatment temperature	SSA (m^2^ g^−1^)*	Pt loading (wt%)	HSR (μg/g min)
INER_A	102 °C	1886	0.35	4.7
INER_B	130 °C	1857	0.11	1.3

^*^SSA: BET determined specific surface area, data quoted from previous studies^6^,^7^,^8^.
