# Supplementary material for: Crystal shape controlled H2 storage rate in nanoporous carbon composite with ultra-fine Pt nanoparticle
Source: Sci Rep. 2017 Feb 14;7:42438. doi: 10.1038/srep42438 (PMC5307954; doi:10.1038/srep42438)
Supplement: Supplementary Information [file srep42438-s1.doc]

**Electronic Supplementary information (ESI) *for***

**Crystal shape controlled H_2_ storage rate in nanoporous carbon composite with ultra-fine Pt nanoparticle**

Tsan-Yao Chen^a,b*^, Yanhui Zhang^c^, Liang-Ching Hsu^d^, Alice Hu^c,*^, Yu Zhuang^c^, Chia-Ming Fan^a,e^, Cheng-Yu Wang^f^, Tsui-Yun Chung^g^, Cheng-Si Tsao^g^, and Haw-Yeu Chuang^h^

^a^ Department of Engineering and System Science, National Tsing Hua University, Hsinchu 30013, Taiwan. E-mail: chencaeser@gmail.com, TEL: +886-3-5715131#34271.

^b^ Institute of Nuclear Engineering and Science, National Tsing Hua University, Hsinchu 30013, Taiwan

^c^ Department of mechanical and biomedical engineering, City University of Hong Kong. Email: alicehu@cityu.edu.hk

^d^ Scientific Research Division, National Synchrotron Radiation Research Center, Hsinchu 300, Taiwan (R.O.C.)

^e^ Industrial Technology Research Institute, Green Energy and Environment Research Laboratories, Hsinchu 310, Taiwan

^f^ Department of Materials Science and Engineering, Feng Chia University, Taichung 40724, Taiwan

^g^ Institute of Nuclear Energy Research, Atomic Energy Council, Executive Yuan, Taoyuan City 32546, Taiwan.

^h^ Green Technology Research Institute, CPC Corporation, Taiwan, Kaohsiung 81126, Taiwan, R.O.C.

*Corresponding author:

Tsan-Yao Chen: email: chencaeser@gmail.com; TEL: +886-3-5715131 ext. 34271; Fax: +886-3-5720724

Alice Hu: email: alicehu@cityu.edu.hk; TEL: +852-34429469; Fax: +852-3442-0172‎

1. Data collection and model analysis on small angle X-ray Scattering spectra of Pt/C NCs.

The small angle X-ray scattering (SAXS) spectra were measured using the Bruker Nanostar instrument. The structure parameters of experimental NCs are obtained by fitting the spectra with scattering model of polydispersed spheres with bimodal Schulz size distribution. The details of calculation program (BimodalSchulzSpheres.ipf) is described in the SANS Model Function Documentation (Version 4.1) which, can be downloaded from the website of National Institute of Standard Technology (NIST NCNR), USA. The size distribution adopted here consists of two Schultz distributions. The nanoparticles in this case have two groups of particles in size. The (normalized) Schulz distribution is^1,2^

where R_avg_ is the mean radius specified by W[1] above and x = R/R_avg_, z is related to the polydispersity, p = σ / R_avg_, by z = 1/p^2^ -1. σ^2^ is the variance of the distribution. The distribution can be plotted for the radius and polydispersity in the coef_bss wave using the marco “Plot_Bimodal_Distribution()”.

The Nth moment of size distribution is equal to

The form factor is normalized by the average volume, using the 3^rd^ moment of R:

The variables w[0] and w[4] are the volume fraction of each of the populations, the total volume fraction is the sum. Since there are two populations, there is no scale factor as in other models. The returned value is the differential macroscopic scattering cross-section (scattered cross-section per unit sample volume per unit solid angle).

Where N_0_ is the total number of particles per unit volume, and Δρ = w[3] – w[8] or w[7] – w[8], is the difference in scattering length density. The number of particles per unit volume having size between R and R+dR is equal to N(R) dR = N_0_ f(R) dR. The Marco Plot_Bimodal_Distribution plots the function f(R) vs R.

2. Estimation on surface to bulk ratio of nanoparticles

🡪 2.1 surface to bulk ratio of spherical particle

From a geometric standpoint, the surface to bulk ratio ($\rho_{S-V}$) for a particle can be estimated by using the following equation:

$\rho_{S-V}=\frac{n_{S}}{n_{t}}$... (S2)

where *n_S_* and *n_t_* denote the number of surface atoms and total number of atoms, respectively. The *n_S_* can be determined by dividing the occupied area ($P_{S}\times S'$, *P_S_* denotes the surface atomic packing factor of a surface, and the surface area of the particle *S'* is equal to $4 \alpha\pi R^{2}$with radius *R*) by the cross section area of particle ($\pi r^{2}$, where *r* denotes the radius of the atom). For a spherical particle, *S'* can be determined by multiplying the surface area of a sphere by a shape modification factor (*α*) that considers the extent of interfacet truncation. Therefore, we can determine n_s_ by using Eq. S3.

$n_{S}=P_{S}\frac{S'}{\pi r^{2}}=P_{S}\frac{4 \alpha\pi R^{2}}{\pi r^{2}}=4 \alpha P_{S}\frac{R^{2}}{r^{2}}$...(S3)

The volume of a particle occupied by atoms (*V_L_*) can be calculated by combining the half volume of the surface atoms ($0.5\times n_{S}\times V_{a}$) and the volume of the interior atoms ($n_{i}\times V_{a}$), where *V_a_* denotes the atom volume. If a particle has a volume of *V_P_*, the occupied volume can be presented as follows by adopting a bulk lattice atomic packing factor, P_L_ (Eq. S4).

$V_{L}=P_{L}\times V_{P}$...(S4)

By combining Eq. (S3) and Eq. (S4), we can obtain the following relationship:

$V_{L}=n_{i}V_{a}+\frac{1}{2}n_{S}V_{a}=P_{L}V_{P}\Rightarrow n_{i}+\frac{1}{2}n_{S}=P_{L}\frac{V_{P}}{V_{a}}=P_{L}\frac{R^{3}}{r^{3}}$...(S5)

Here, the total number of atoms in a particle can be represented as $n_{t}=n_{i}+n_{S}$. According to Eq. (S3) and Eq. (S5), *n_t_* can be represented as:

$n_{t}=P_{L}\frac{R^{3}}{r^{3}}+2 \alpha P_{S}\frac{R^{2}}{r^{2}}$...(S6)

therefore, $\rho_{S-V}$ is obtained from the following numerical representation:

$\frac{n_{S}}{n_{t}}=\frac{4 \alpha P_{S}\frac{R^{2}}{r^{2}}}{P_{L}\frac{R^{3}}{r^{3}}+2 \alpha P_{S}\frac{R^{2}}{r^{2}}}=\frac{4 \alpha P_{S}}{P_{L}\frac{R}{r}+2 \alpha P_{S}}$...(S7)

Because the shape factor parameters and the packing factors are dominated by particle shape, $\rho_{S-V}$ is inversely proportional to the radius of the particle (R).

- 2.2 surface to bulk ratio of disk-like particle

Regardless of the shape of particle, the surface to bulk ratio ($\rho_{S-V}$) for a disk-like particle is estimated by equation S2; where corresponding n_s_ and n_t_ are determined considering to the associating shape factor and the derivations are shown in following equations. In these equation, S is surface area, D is diameter, H is thickness, and Vp is volume of the disk particle. In addition, d is the diameter and Va is volume of atom.


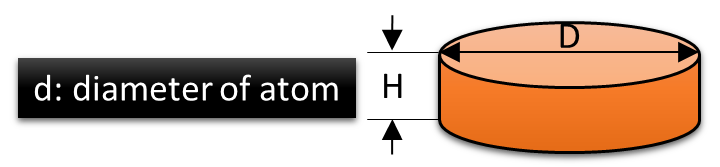

$$\begin{matrix} S= 2\times\pi(\frac{D}{2})^{2}+2\pi(\frac{D}{2})H \\ V_{P}=\pi(\frac{D}{2})^{2}H \end{matrix}$$

$\frac{n_{S}}{n_{t}}=\frac{2P_{S}(\frac{1}{H}+\frac{2}{D})}{\frac{3}{2}P_{L}+P_{S}d(\frac{1}{H}+\frac{2}{D})}$…(S8)

$P_{L}\times V_{P}=n_{i}\times V_{a}+\frac{1}{2}n_{S}\times V_{a}\Rightarrow$…(S9)

$n_{i}+\frac{1}{2}n_{S}=P_{L}\frac{V_{P}}{V_{a}}=P_{L}\frac{\pi D^{2}H/4}{\pi d^{3}/6}=\frac{3}{2}P_{L}H\frac{D^{2}}{d^{3}}$…(S10)

In these equations, n_i_ is the number of interior atom in the particle. P_L_ (volume atomic packing density of bulk region) x V_P_ is the total volume of bulk (which without exposing to ambient). This value is represented by sum of volume of bulk (n_i_ x V_a_) and half volume of the shell region (which exposing to bulk region) from the outmost region of particle (1/2 n_s_ x V_a_). The number of atoms in surface region is determined by total surface area that occupied by atoms (i.e., P_S_ (surface atomic packing density) x A_total_) divided by the cross section of atom (eqn S9).

$n_{S}=P_{S}\frac{\pi DH+2\pi(\frac{D}{2})^{2}}{\pi(\frac{d}{2})^{2}}=2P_{S}(2\frac{DH}{d^{2}}+\frac{D^{2}}{d^{2}})$…(S11)

In this event, total atoms in the particle is represented by eqn S10

$\therefore n_{t}=n_{i}+n_{S}=\frac{3}{2}P_{L}H\frac{D^{2}}{d^{3}}+P_{S}(2\frac{DH}{d^{2}}+\frac{D^{2}}{d^{2}})$…(S12)

- 2.3 surface to bulk ratio of disk-like particle octahedron particle


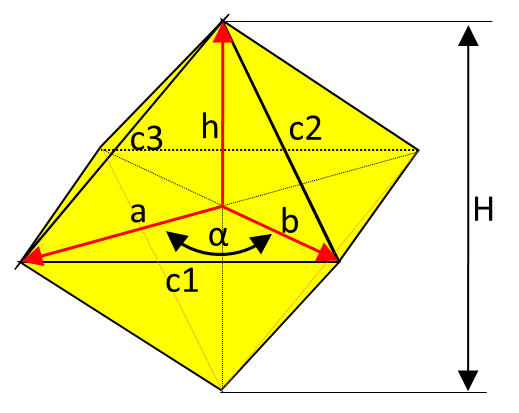


For octahedron particle, the surface to bulk ratio is determined according to eqn S9:

$\frac{n_{S}}{n_{t}}=\frac{\frac{32\Delta}{\pi d^{2}}}{P_{L}\frac{16A\times h}{\pi d^{3}}+P_{S}\frac{16\Delta}{\pi d^{2}}}=\frac{2d\Delta}{P_{L}A\times h+P_{S}d\Delta}$…(S13)

where

$n_{S}=P_{S}\frac{S}{\pi(\frac{d}{2})^{2}}=P_{S}\frac{32\Delta}{\pi d^{2}}$…(S14)

Δ is the area of single triangle surface of octahedron particle, d is diameter of atom.

$n_{t}=(n_{i} +\frac{1}{2}n_{S})+\frac{1}{2}n_{S}=P_{L}\frac{16A\times h}{\pi d^{3}}+P_{S}\frac{16\Delta}{\pi d^{2}}$…(S15)

A = ½ x a x b x sin (180 – α) is the area of triangle in horizontal plan of octahedron (which is one quarter of the horizontal plan) and V_P_ (= 8 x 1/3 x A x h) is the volume of octahedron particle (h is the half height of octahedron). P_L_ x V_P_ is the volume of particle and is represented in the same form of disk particle (S9 and 16). With the geometrical consideration, the total atoms of octahedron particle (n_i_ + 1/2n_s_) can be estimated by the corresponding volume been occupied by atoms (i.e., volume of particle multiplied by volume atomic packing density and divided by volume of atom in eqn S17)

$P_{L}V_{P}=n_{i} V_{a}+\frac{1}{2}n_{S} V_{a}$…(S16)

$n_{i} +\frac{1}{2}n_{S} =P_{L}\frac{V_{P}}{V_{a}}=P_{L}\frac{8\times\frac{1}{3}A\times h}{\frac{4}{3}\pi(\frac{d}{2})^{3}}=P_{L}\frac{16A\times h}{\pi d^{3}}$…(S17)

3. XAS analysis of carbon supported Pt clusters


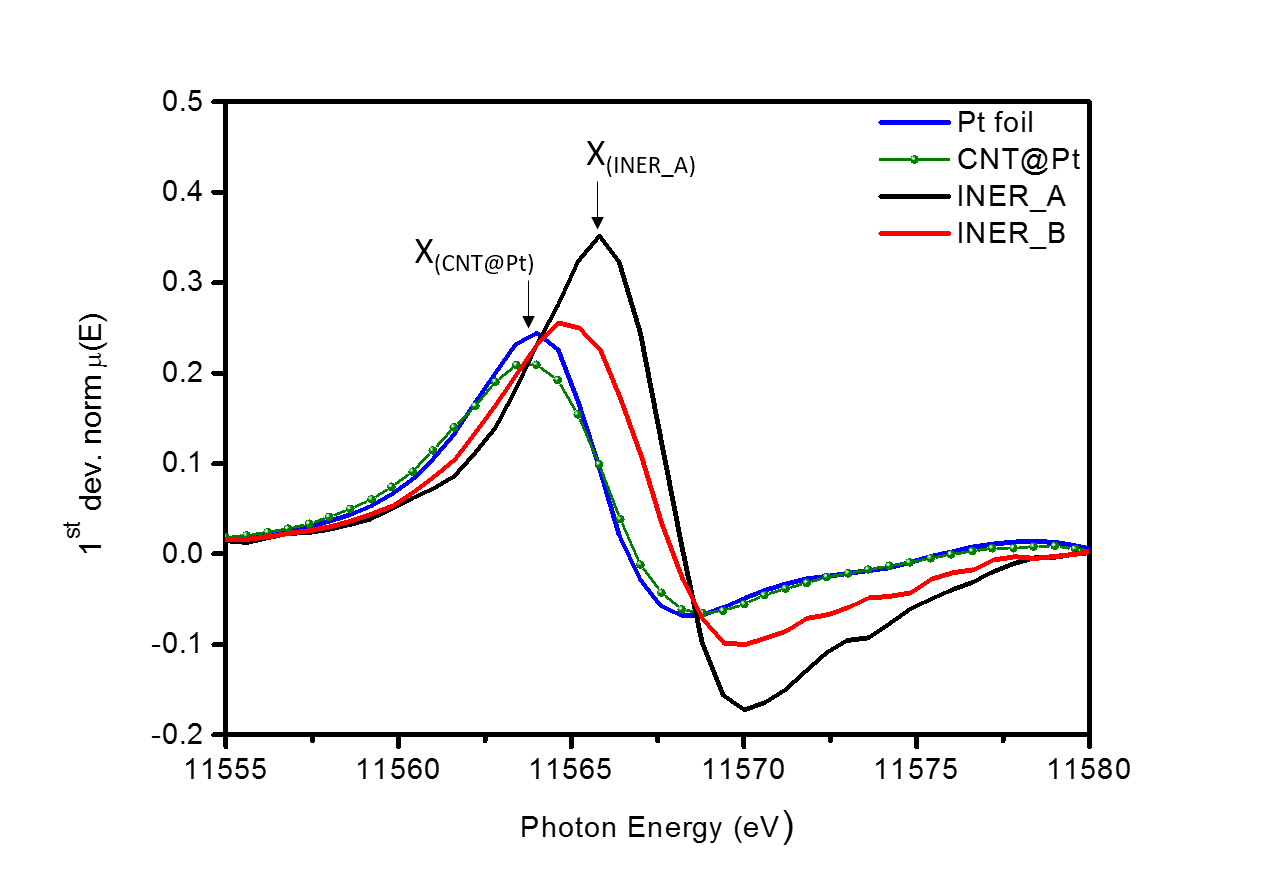


Fig. S1 1^st^ deviation of X-ray absorption near-edge spectra for carbon supported Pt cluster samples

4. DFT simulations on Pt positions in graphite surface


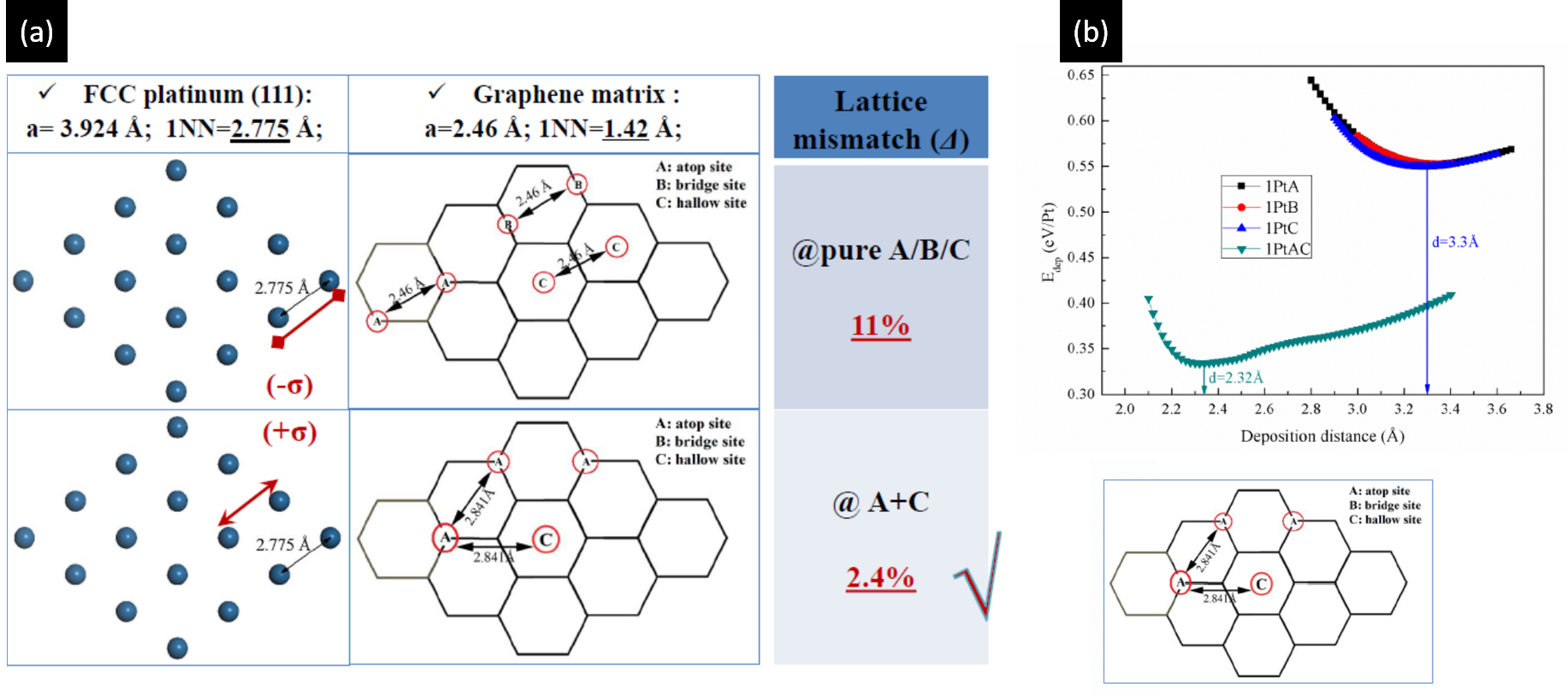


Fig. S2 (a) atomic structure models of single layer Pt atoms in different sites of single layer graphite surface. Scheme in top shows models of Pt atoms in top of atop (1PtA), bridge (1PtB), and hollow sites (1PtC) of graphite surface. Scheme in bottom shows model of Pt atoms pairing in atop and hollow sites of graphite surface (1PtAC). (b) corresponding energy profiles of the models upon Pt relaxation to the optimum state.

According to Fig. S2b, model consisted of two Pt atoms pairing at atop and hollow sites in carbon structure showing the lowest relative energy at the final energy state (d = 2.32 Å) among all models.

Reference

1 Kotlarchyk, M. & Chen, S.-H. Analysis of small angle neutron scattering spectra from polydisperse interacting colloids. *The Journal of Chemical Physics* **79**, 2461-2469, doi:10.1063/1.446055 (1983).

2 Schulz, G. V. The kinetics of chain polymerization. V. The effect of various reaction species on the multimolecularity *Z. Phys. Chem.* **B43**, 25-46 (1935).
